# The Role of International Missions in Advancing Cardiac Surgery in Africa

**DOI:** 10.21470/1678-9741-2025-0266

**Published:** 2025-12-10

**Authors:** Adnaldo da Silveira Maia

**Affiliations:** 1 Cirurgia Cardiovascular, Universidade de São Paulo, Instituto do Coração, São Paulo, São Paulo, Brazil. E-mail: adsm.ccv@gmail.com

The challenge of performing cardiac surgery in sub-Saharan Africa remains substantial. With a limited number of specialized centers across the region, many patients await surgical intervention at advanced stages of disease. Among the available strategies, in addition to governmental support, the establishment of international missions has played a pivotal role in recent years in developing and empowering local surgical teams^[1]^.

The work by Nyawawa et al.^[1]^ exemplifies this progressive development. The authors report the outcomes achieved by the local team at the Jakaya Kikwete Cardiac Institute in Tanzania, following several international missions, in patients undergoing coronary artery bypass grafting. Among the 290 patients operated on, 131 (45.2%) procedures were performed by the local team. The analysis revealed longer cardiopulmonary bypass and aortic cross-clamping times in surgeries conducted by the local group (*P* = 0.001). However, no statistically significant differences in mortality were observed between groups, with outcomes comparable to those reported in other countries. Additionally, the study documented an increasing number of cases across various missions (Saifee, Apollo, Open Heart International, CardioStart, World Regent, Egyptian Mission), with more than 80% of patients recovering postoperatively, highlighting the commitment and resilience of the teams involved^[1]^.

## The Burden of Cardiovascular Disease in Africa

Coronary artery disease remains the leading cause of death worldwide. However, two groups of diseases account for a substantial portion of the need for cardiac surgery in low-income countries: congenital heart disease and rheumatic heart disease (RHD). The statistics are striking. Countries such as Mozambique report approximately seven cardiac surgeries per million inhabitants, Nigeria only 0.5, Namibia reports 127, and South Africa reports 5,300, in stark contrast to high-income countries such as Germany (41,190) and Brazil (8,129). Similarly, the availability of cardiac surgeons remains a critical challenge, with ratios as low as 0.2 per million inhabitants in Mozambique and 0.06 in Nigeria^[2]^.

Karthikeyan et al.^[3]^, in an evaluation of 13,696 patients from 24 lowand middle-income countries, demonstrated the significant impact of RHD-related mortality, with approximately 15% of patients dying within the first three years of follow-up. Despite the majority being symptomatic, only about 5% underwent valve surgery.

In this context, humanitarian missions have played a crucial role in supporting the development of local cardiac teams around the world. Several organizations, including CardioStart International, the VOOM Foundation, and Heart to Heart, among others, have contributed through the voluntary efforts of professionals who donate their time and expertise to patient care. Nevertheless, the active involvement and training of local teams are the cornerstone of these missions' long-term impact, as fostering autonomy among local providers ensures continuity of care after the missions conclude^[3]^.

Nina et al.^[4]^ describe that the primary goal of any mission should be to provide training to local teams. An educational program model in this context should include a few essential steps:

• Site selection
• Demographic assessment
• Site analysis
• Team organization
• Regularly scheduled missions
• Program development monitoring and maturation analysis

**Fig. 1:**
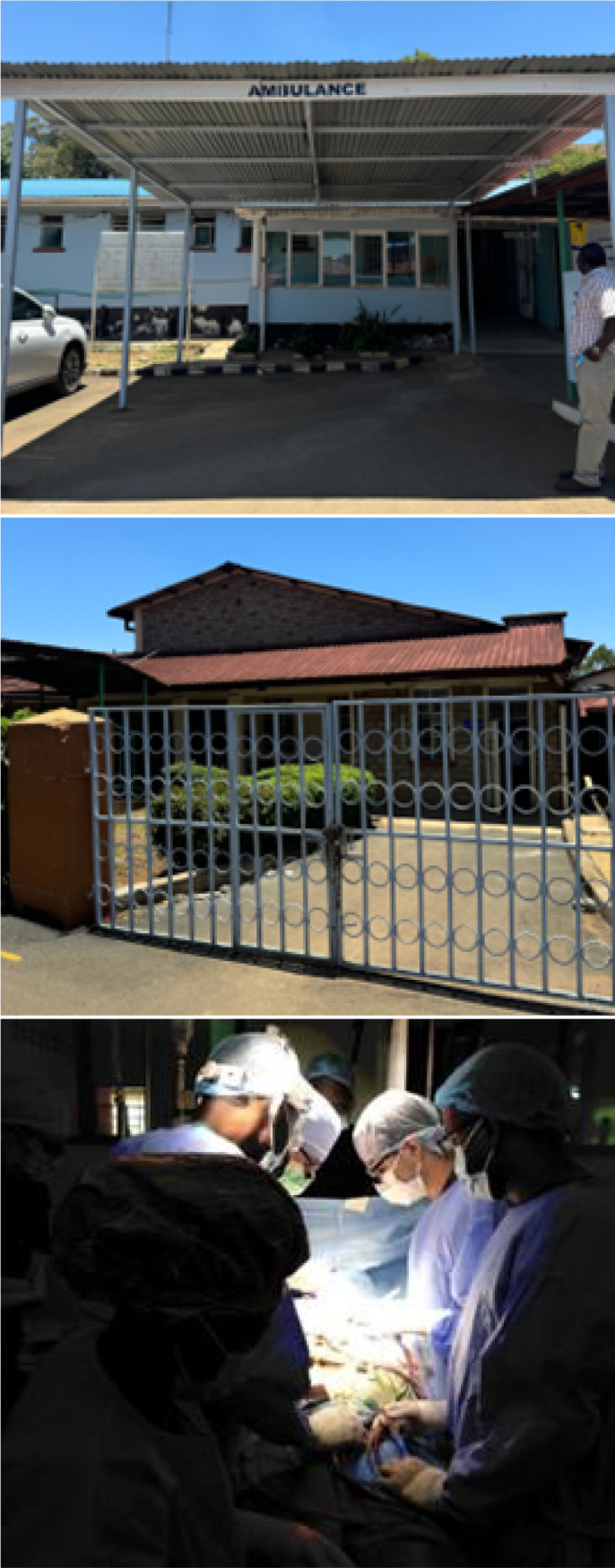
International Mission in Cardiac Surgery at Moi Teaching & Referral Hospital (MTRH), Eldoret, Kenya.

In addition to international organizations, partnerships with universities represent another viable pathway for establishing training programs in cardiac surgery. One such example is the Academic Model Providing Access to Healthcare (AMPATH), a partnership between Indiana University, Moi University, and other academic institutions based in the United States of America, established in 2001. Today, AMPATH supports a population of more than 24 million in Western Kenya, across over 300 sites, with referrals directed to Moi Teaching and Referral Hospital^[4^,^5]^. Grants offered by the Thoracic Surgery Foundation, the charitable arm of the Society of Thoracic Surgeons, provide another avenue for participation and engagement, enabling cardiac surgeons, residents, and fellows from different countries to contribute to local team training efforts (Figure 1).

The support from various international organizations directly contributed to the outcomes reported by Nyawawa et al.^[1]^ in the treatment of patients with coronary artery disease. The technical development of the local team throughout the missions - supported by observerships in Israel, the United States of America, and Brazil - enabled procedures to be performed locally that previously required referral abroad. The authors’ retrospective analysis reinforces the value of such efforts and serves as an inspiration to other teams worldwide.

The experience of international missions in Africa illustrates not only the urgent need for expanded access to cardiac surgical care but also the transformative potential of collaborative, education-focused efforts. While humanitarian interventions initially serve to address immediate surgical demands, their true value lies in fostering sustainable local capacity. The example of Jakaya Kikwete Cardiac Institute and other similar initiatives highlights the importance of structured training, long-term partnerships, and the commitment of both local and international teams. To reduce the burden of untreated cardiovascular disease in low-resource settings, future strategies must prioritize education, autonomy, and systems strengthening - ensuring that cardiac surgery becomes a lasting and locally driven reality across the African continent.

## Artificial Intelligence Usage

The author declares that no artificial intelligence tool was used in the preparation of this article.
